# Supplementary material for: Investigating the welfare and conservation implications of alligator wrestling for American Alligators (Alligator mississippiensis)
Source: PLoS One. 2020 Nov 13;15(11):e0242106. doi: 10.1371/journal.pone.0242106 (PMC7665580; doi:10.1371/journal.pone.0242106)
Supplement: Attachment — Submitted filename: Response to Reviewers.docx [file pone.0242106.s002.docx]

OCTOBER 8, 2020

Dear Editorial Board,

Thank you for the reviews of our manuscript “Investigating the welfare and conservation implications of alligator wrestling for American Alligators (*Alligator mississippiensis*).” We have revised portions of our qualitative analyses and discussion to strengthen our argument and align more closely with the available research on the conservation value of wildlife tourism attractions. We have also updated the reporting of some percentages, added additional references to support specific claims, and adjusted terminology to maintain consistency throughout the text. In making these changes, we believe the paper to be much improved, strengthening its potential contribution to the literature. We wish to thank the reviewers for their careful attention to our work and for their ideas and insights regarding its potential.

Below please find our detailed responses to their comments as well as indications to how we have revised the manuscript. The reviewer and editorial comments are in grey Times New Roman font, our responses are in black Calibri light font, and updated passages from the manuscript are in red Calibri light font. All changes to the manuscript itself have been noted with “track-changes” and all line numbers in this response letter refer to the line of the updated manuscript when viewed through the “All Markup” option in track-changes. We have also included a clean copy of the revised manuscript in our submission.

The data reported in this manuscript have not been published previously and are not under consideration for publication elsewhere. All listed authors have contributed significantly to the manuscript and consent to their names on the manuscript. Each author declares no competing financial interests and affirms that she has read the PLOS ONE journal requirements on author responsibilities and is submitting the manuscript in accordance with those policies. Finally, we have amended our manuscript to include an ethics statement in compliance with the YouTube Terms of Service, per the requirements for resubmission.

Thank you again for your consideration, and we look forward to hearing from you regarding the status of our manuscript.

Sincerely,

Dr. Becca Franks (Corresponding author; [krf205@nyu.edu](mailto:krf205@nyu.edu))

Casey Riordan, MSc, MA (Coauthor; cjr524@nyu.edu)

Dr. Jennifer Jacquet (Coauthor; jacquet@nyu.edu)

Editor:

Thank you for submitting your manuscript to PLOS ONE. After careful consideration, we feel that it has merit but does not fully meet PLOS ONE’s publication criteria as it currently stands. Therefore, we invite you to submit a revised version of the manuscript that addresses the points raised during the review process.

We wish to thank the Editor and the reviewers for their encouragement about the importance of this topic and for their helpful suggestions for ways to improve and strengthen our manuscript. In response to their commentary, we have incorporated the requested references, provided additional context where needed for our methodology and analysis, and ensured that our language is more consistent throughout the document. All changes to our manuscript have been tracked and all line numbers in this response letter refer to the line of the updated, tracked manuscript viewed through the “All Markup” setting. In re-submitting our paper for consideration, please note that we have also ensured that the document meets the PLOS ONE style requirements and incorporated a YouTube compliance statement in the methodology section in lines 225-228.

Reviewer 1:

R1.0) Authors analysed videos of alligator wrestling from YouTube and evaluated welfare and conservation matters. Results showed that the reptiles presented a low welfare and that no conservation message is being passed by the wrestlers. The paper is very interesting, brings novelty to the scientific literature, and proposes a modern method for this type of study. For this, the study should be considered for publication. However, some revision in needed. See the commentaries bellow:

We wish to thank the reviewer for their supportive comments regarding the value of our manuscript and our choice of methodology. We are grateful for the suggestions made to help us improve the quality of the study. All changes to the manuscript itself have been tracked using the “track changes” setting on Microsoft Word, and all line numbers in this response refer to the line of the updated manuscript in the “All Markup” setting.

R1.1) Line 54: insert a reference after 'literature'

We have inserted the appropriate references after ‘literature’ in line 73.

R1.2) Line 72: insert a reference after 'behaviors'

We have inserted the appropriate reference after ‘behaviors’ in line 92.

R1.3) Line 85: insert a reference after 'United States'

We acknowledge that the sentence in question, “The American Alligator (*Alligator mississippiensis*) is one of the most commonly used reptiles in WTAs in the United States,” may come across as overstated without further qualification. As such, we have revised it to the following sentence, justified by the nature of our study (beginning line 123):

Thank you for pointing out the need to include a reference at the end of this statement. Upon careful examination of the evidence, it turns out that no systematic evaluation of all the reptiles used in WTAs in the US has been conducted. As such, we have adjusted the sentence to read as follows (beginning line 103):

The American Alligator (*Alligator mississippiensis*) is one of the few reptiles commonly found in WTAs in the United States.

R1.4) Line 126: insert a reference after 'education'

We have inserted the appropriate references after ‘education’ in line 157.

R1.5) Line 140: insert a reference after 'facility'

Thank you for pointing this out. Although there have been numerous news articles written about nuisance alligators being relocated to captive facilities (for example see Naples Daily News, 2019; Pulptown, 2019; Tampa Bay Times, 2018), the FWC only publishes official statewide data on nuisance alligator harvests. As such, we have revised the sentence to the following, along with the appropriate reference (beginning line 182):

Instead, alligators deemed a nuisance due to their size and location near humans and domestic animals are often slaughtered, with 7,669 nuisance alligators harvested out of 14,072 permits issued for alligator removals in 2019.

R1.6) Line 194: provide the criteria to classify each venue as large, medium or small. This criteria is already in lines 248-254, but it is better to remove them to line 194.

Thank you very much for this recommendation, we agree it would be helpful to elaborate on venue sizes earlier on in the manuscript. Per this suggestion we have listed the criteria starting in line 298 of the methodology section, and based on feedback from other reviewers, we have also converted the measurements to the SI system and included an explanation for how we went about estimating the size of each venue.

R1.7) Lines 261-263: what about the 16% remainder cases?

The remainder of cases were unclear, either because the video contained multiple clips of separate alligator wrestling shows or because the water feature was too murky to discern other alligators present. We have made this more explicit in the text, starting in line 449.

R1.8) Lines 439-440: but the mean wrestling time was 5 minutes and the majority of the wrestkers are not performing the stressing behaviors.

Thank you for pointing this out, and we agree that this statement needs clarification. The original study by Franklin et al. (2003) found that crocodilians who were manually restrained for *up to* 10 minutes took 8 hours for their blood stress levels to reduce, and that it took anywhere from 1.6 to 15.5 minutes to capture, restrain, measure, and return the alligators back to their enclosure (p. 88). Under these conditions, the restraint times present in our dataset are potentially stressful for the alligators. We have edited the sentences starting in line 755 to reflect this:

An additional study found that physically restraining crocodilians is more stressful for them than using a stun gun, and that it can take up to eight hours for their blood stress levels to reduce after being physically restrained between two and 15 minutes [60]. Our study revealed that 96% of wrestlers restrained alligators in their performances, and that the median restraint time for videos that showed the entire duration of restraint was five minutes. This indicates that many wrestled alligators are being restrained long enough to heighten their blood stress levels.

R1.9) Lines 451-467: it is important to separate the maintenance enclosures from the show enclosures. To keep more animals together in the show enclosure is more attractive for the audience. An evaluation of the maintenance enclosures showed be conducted to see if the animals are being hold together. This, in fact, was suggested by the authors at the 'Future research' topic.

Yes, this is indeed a very good point, and we did not make a strong enough distinction in our discussion of the environmental threats of performance arenas versus maintenance arenas. To address this, we have made several changes. First, we edited the text starting in line 771 to the following: “In addition to the potentially stressful behaviors inflicted upon wrestled alligators, it is likely that some of the environmental features present in the performance arenas may pose welfare threats to wrestled alligators.” Secondly, we elaborated on the distinction between maintenance enclosures vs. performance arenas starting in line 790:

It is worth noting that some wrestlers bring wrestled alligators into a temporary arena for performances, after which they are released back into a maintenance enclosure, that may or may not contain water features that meet the requirements set out by law. Based on the limited nature of YouTube data, this study was only able to consider performance arenas, and while certain performance arenas appeared to double as maintenance enclosures, it was unclear the extent to which this was the case in the data set.

R1.10) Lines 468-476: the total time of exposure to humans should be considered to evaluate if the animals are suffering from acute or chronic stress.

Thank you for this interesting suggestion. We agree that total exposure time to humans is an important variable. Unfortunately, with the current data set, we are unable to gauge total time as the clips always contain humans, but don’t constitute a random sample from a typical day in the life of one of these alligators. Thus, getting an overall estimate of human exposure is impossible. We can infer minimum exposure time, however, with individual video length. As this value is such an obvious underestimate of likely exposure, we do not believe that reporting it as such would enhance the impact of the MS. At the Editor’s discretion, therefore, we would prefer to remain silent on this important, but as yet, unknown variable.

R1.11) Line 496: insert the year of the study of 'Moorhouse et al.'

We have added the year of the Moorhouse et al. study to line 874.

R1.12) Lines 550-558: It is a good study method, but it can input errors in the analysis, as atated by the authors, due to the small duration of the videos. How many videos had the entire recording of the wrestiling show? Please, inform this here.

This is an important consideration and will benefit from added precision in the limitations/weaknesses section. Thank you for encouraging us to report this number, which we have incorporated into lines 1033-1036.

R1.13) Line 578: to interview the audience could bring information about the conservation message learned during the shows.

We have incorporated this suggestion into our future research opportunity section, starting in line 1051.

Reviewer 2:

R2.0) Wildlife tourism attractions (WTA) with alligators are popular in Florida but the authors report that only two research studies have previously investigated these activities. The present study therefore wants to fill this gap. The analysis took into consideration 94 YouTube videos to investigate what happens during alligator wrestling and to collect data that can give indications about the welfare of the animals involved and the conservation implications of such activities. The authors found that in WTA classified as "alligator wrestling" animals are subjected to interactions that do not guarantee their welfare. Furthermore, the analysis of the contents of the wrestlers’ narrations revealed that they are mainly for recreational purposes and only superficially deal with conservation issues. The manuscript is technically sound, and the data support the conclusions. The position of the authors is therefore justified when they say that even if wrestling activities were accompanied by valid educational and conservationist messages, they would still have doubts that wrestling in itself can be an effective way to promote a positive relationship between animals and humans. It is therefore ethically sound that the authors have chosen a data collection method that does not feed these types of activities.

We appreciate the reviewer’s support of our method and for their thoughtful suggestions. We have adjusted the document per their suggestions, with specific changes detailed below. All changes in the document itself have been done using the “track changes” feature of Microsoft Word, and all line numbers in this response letter refer to the line of the updated manuscript when viewed through the “All Markup” setting.

R2.1) Lines 86, 250, 252, 254. Pounds, feet, and yards should be replaced with the units of the international system of Units (SI).

We have converted all references to pounds, feet, and yards to the units of the SI.

R2.2) Line 143 Not all authors agree that keeping alligators in captivity is useless to protect wild populations. Among the conservation actions reported in the IUCN Red List is included the following paragraph. “Because of the extensive commercial ranching and farming industry in the USA, the American alligator is a prime candidate for research on captive husbandry. Incubation and rearing techniques need to be improved to increase the efficiency of the alligator ranching industry. Extensive research on these topics is currently underway, particularly in Louisiana (Reigh and Williams 2016) and Florida.” (Elsey, R., Woodward, A. & Balaguera-Reina, S.A. 2019. Alligator mississippiensis. The IUCN Red List of Threatened Species 2019: e.T46583A3009637. <https://dx.doi.org/10.2305/IUCN.UK.2019-2.RLTS.T46583A3009637.en>.

Thank you very much for bringing these recommended actions to our attention. We acknowledge that some bodies have indicated that keeping alligators in captivity may provide some indirect conservation benefits, especially when it offers opportunities for research. Our goal in this passage was to emphasize that the mere act of keeping alligators in captivity does not currently provide a *direct* benefit to the species in terms of maintaining overall population levels. We have fixed our wording of this statement (starting in line 188) to make this distinction. Furthermore, although captive alligator research may be important to current and future conservation efforts, we would argue that it is unclear whether researching captive alligators used for wrestling, where the environmental conditions and stressful behaviors inflicted upon these animals are highly unnatural, would produce insights that can accurately inform wild and captive conservation efforts. We have made a note of this in the text, starting in line 189.

R2.3) Line 229 - 232 Although native facilities have cultural and historical justifications, from an ethical point of view, this is not sufficient to derogate from minimum animal welfare standards. Moreover, given the increasingly severe environmental problems throughout the planet, the use of animals should always be accompanied by a commitment to conservation and the promotion of respect for nature.

We wholeheartedly agree, and it is our hope that growing environmental concerns coupled with increased awareness of the harms of WTAs will make it apparent that increased industry standards are necessary. The primary reason we excluded native facilities based on cultural and historical justifications is because promoting local cultures and history has been cited on numerous occasions as an acceptable justification for WTAs (Moorhouse, D’Cruze, & Macdonald, 2017; Jones, 2015; Russell & Wallace, 2004). Thus, we felt it was best to consider only the non-native venues who frequently claim that environmental conservation underlies their wrestling attraction. However, we do not want to overlook the importance of this ethical stance, so we have added the following statement beginning line 349:

Though we did not consider native attractions for this portion of the study, it is important to note that having minimum animal welfare and conservation standards should be important ethical considerations for all wrestling attractions moving forward, especially amid the increasing global environmental concerns.

R2.4) Line 250. It is not clear what the enclosure size is. It would seem that "large" is an enclosure of at least 28 sqm (a circle with a radius of 4.57 m), "medium" from 11 to 28 sqm; "small" less than 11 sqm. It is also not clear how the authors arrived at this estimate.

We have converted the measurements to sqm in the manuscript, and per the suggestions of another reviewer we have elaborated on the description of venue sizes (which we have called “arena sizes” in the text per another reviewer recommendation) and how we arrived at our estimates within the methodology section, beginning line 298:

We defined “large arenas” as those with at least 28sqm of space for the alligator within the enclosure. “Medium arenas” provided a more restricted area of approximately 11 to 28sqm of room to move. “Small arenas” were those with approximately 11 sqm of room or less. To classify arena dimensions, we first calculated the mean length of an adult alligator at 3 meters, based on the average length of female alligators (2.6 meters) and male alligators (3.4 meters) [84]. From there, we then estimated the arena sizes in relation to the body length of alligators in each enclosure.

R2.5) Lines 325 and 332. Instructions on how to avoid contact with wild alligators and not to feed them are messages aimed at improving interactions between humans and animals. Therefore, not only can they be considered conservation messages, but since they are explicit indications on which behaviors to adopt or not to adopt, they respond to the indications provided by the study of Ballantyne et al. (2019) (see comments below for lines 488 -491).

Thank you for highlighting this, we did not make it clear enough in this portion of the text that the wrestlers who provided instructions about avoiding wild alligators were offering both practical *and* conservation-driven messaging. We have amended lines 547-550 as well as lines 855-857 to make this more apparent. We have also addressed the comment made later on about our alignment with the Ballantyne et al. (2009) study (please refer to the comment labeled R2.9 for specific details of our changes).

R2.6) Line 342 The fact that a species is classified as a least concern does not guarantee that the species will not decline in the future even in the short term. There are examples in the history of widespread species that have been extinct in a short time (e.g., Ectopistes migratorius). The same species Alligator mississippiensis was classified as endangered in the 1960s. Today the species is not at risk because of the success of the management and conservation plans implemented. It could be an example of sustainable management despite withdrawals.

This is a great point, and we agree that there is arguably conservation value in sustainably managing captive populations to protect against future declines. Here, we aimed to convey that without specifying the actions being taken to manage their populations in a sustainable and humane way in accordance with statewide conservation goals, it is unclear whether these attractions are truly an effective source of conservation (versus simply providing animal welfare value, which again is dubious given their treatment of the alligators wrestled in their care). We have amended the text beginning in line 562 to take this suggestion into account, and to elaborate on our argument:

The second sub-theme under environmental awareness was alligator protection. For example, some wrestlers stated that their facility rescues and rehabilitates wild nuisance alligators or informed the audience that their donations support the upkeep of captive alligators in their venue. However, given the treatment of alligators who are repeatedly wrestled at these facilities, it is unclear whether rescuing nuisance alligators from the wild and placing them into captive wrestling venues is more preferable from a welfare standpoint than killing nuisance alligators, which is the standard protocol set forth in Florida’s nuisance alligator management program [73]. Furthermore, from a conservation perspective, not one wrestler explicitly addressed how their rescue and rehabilitation efforts support overall alligator conservation goals. This is important because alligators were previously listed as an endangered species from 1967 until 1987, when populations recovered largely due to successful conservation programs [90]. Though wild populations are currently thriving enough to make captive management arguably unnecessary to directly sustain overall population levels [21], maintaining captive alligator populations may help to mitigate against future declines if their captive maintenance incorporates opportunities for the natural behaviors and experiences that would be necessary for population survival. However, without specifying the actions that alligator wrestling attractions are taking to sustainably and responsibly manage their alligators in accordance with statewide conservation goals, the conservation value of these attractions remains unclear. Finally, none of the commentators noted whether their alligator wrestling attraction makes a monetary donation toward external conservation or protection initiatives, which may be another way to benefit ongoing conservation efforts.

R2.7) Line 347 and line 516 Talking about the anatomy of the species could be a way to introduce animal welfare issues, so it could still be a useful message even if not strictly related to species conservation.

We have amended the text beginning in line 602 to note that the miscellaneous educational comments, while not directly related to environmental or welfare issues, may potentially facilitate a future discussion of alligator conservation and protection. This is also something we have noted later on in the manuscript (beginning line 841) along with a reference to Ballantyne et al. (2011), who found that providing facts about wild animals at wildlife attractions may not be as memorable for tourists than other conservation messaging, such as providing practical advice for supporting conservation.

R2.8) Lines 471 - 473 Although it is undeniable that zoo animals may be subject to some stress due to noise, public proximity, or other factors. When comparing animals housed in zoos and those used in wrestling with alligators, it would be right to also highlight the differences between the two types of management.

We agree that there are undoubtedly managerial differences between zoos/aquariums and wrestling facilities. Rather than contrasting these differences, which we feel may be rather speculative at this stage, we have opted instead to alter the statement to remove the explicit reference to zoos. It now reads as follows (beginning line 812):

Enduring human contact is not unique to wrestled alligators, as it is a common stressor imposed upon nearly every animal kept in captivity at wildlife tourist attractions, even those with strict animal welfare standards [25]. However, given the added stressors experienced by alligators who are repeatedly used for wrestling performances, it may be of particular importance for all wrestled alligators to have (at a minimum) access to murky water or waterside vegetation to conceal themselves similarly to how they would in the wild.

R2.9) Line 488- 491 Quoting Ballantyne et al. (2009), authors affirm that “Among wrestlers who discussed environmental topics, none discussed the role of alligators in their natural habitats, which has been cited as an important component of conservation education as it helps visitors understand why protecting these animals is needed”. But it seems that the core message of the article cited is different: “Tourists were particularly interested in practical information about what they could do to help protect the wildlife, rather than general information about conservation issues.” (pag. 663) and “The findings of this study suggest that the key to balancing the needs of tourists with the needs of wildlife, is to clearly communicate the reasons behind particular management practices in terms that relate directly to protecting the animals from human impacts.” (pag.663)

Thank you for pointing this out. We agree that the manuscript does not align closely enough with the Ballantyne et al. (2009) study. We have edited the text starting in line 547 to specify that some wrestling attractions did, indeed, offer practical tips for tourists to support conservation initiatives. We have also attempted to make it clearer beginning in line 898 that many wrestling attractions did not clearly communicate their conservation value, nor the reasoning behind some of their practices that could potentially come across as misleading or contradictory; this in turn may confuse visitors and can even undermine the educational value of these attractions.

R2.10) Lines 540 - 543 Furthermore, from an ethical point of view, claiming to promote the conservation of the species without clarifying what actions are taken by the various venues is also a limitation for visitors. In fact, visitors do not have the necessary information to understand whether the economic contribution they make by paying for the ticket really benefits conservation and how.

This is a very helpful point, thank you for bringing it to our attention. We have incorporated it into our discussion, starting in line 913.

Reviewer 3:

Abstract
R3.0) Line 30: Remove ‘of’ between ‘94’ and ‘YouTube’

We have amended this sentence in line 29.

R3.1) Line 31: Why ‘at least 15 venues’? Can you be more accurate? Later, in table 1 (line 197), 16 venues are listed.

Although we categorized 16 venues, the sixteenth category includes “unnamed Florida locations,” meaning venues that were noted as being in Florida but did not specify the name of the wrestling venue. Therefore, when we stated that 11 out of 15 venues housed adult alligators together with conspecifics, we were only referring to the 15 discernible venues in the dataset. To avoid confusing readers, we have amended line 30 and line 33 to remove references to 15 venues.

R3.2) Otherwise, a very good abstract.

We wish to thank the reviewer for these very productive suggestions to improve our abstract and the remainder of our manuscript, which were particularly helpful for making the text more consistent and accurate. We have adjusted the document per their suggestions, with specific changes detailed below. All changes in the document itself have been done using the “track changes” feature of Microsoft Word, and all line numbers in this response letter refer to the line of the updated manuscript when viewed through the “All Markup” setting.

Introduction:
R3.3) This section describes categories of WTA and potential impacts on the wildlife before introducing the understudied alligator and the history of wrestling. Final paragraph explains what the study has done. Here an explanation of the overarching aim could be more clearly made. As reference is made later to numbered research questions, see below, are they missing from here?

Thank you very much for pointing this out. We have clarified in the final paragraph (starting in line 196) that the overarching aim of the study is to investigate and evaluate the environmental and animal welfare claims made at alligator wrestling venues.

Materials and Methods:
R3.4) Line 186: “To answer research questions 1 and 2 ..” The reader has not yet been told what these are (see comment connected to the missing aim from introduction).

We are very grateful that you pointed out this confusion in the text, as this phrasing was taken from an earlier version of the draft and was intended to be removed from the final manuscript. We have amended the text beginning line 270 to remove the reference to research questions, and to clarify that we watched each video twice in order to comprehensively assess the environmental and behavioral characteristics of alligator wrestling matches.

R3.5) Line 205: Alligator in this sentence should be singular – remove the ‘s’.

We have removed the ‘s’ from alligator in line 320.

Content Analysis:
R3.6) Line 233: the reader is told here that the transcription happened ‘by hand’. What does this actually mean? Date of the transcriptions is included here but does not seem relevant as findings should not change if transcription happened at another time. Relevant is when filming of the videos took place, which is information you have provided.

Per this suggestion, we have removed the dates of transcription in line 354. We have addressed the comment about transcribing by hand in more detail below.

R3.7) Line 235: Similar to above, the reader is told that the analysis happened ‘by hand’. What does this mean? Presumably you mean a software package, such as NVivo, was not utilized. It may be useful to explain why you choose to identify themes manually.

Yes, this is correct: We transcribed and analyzed the videos manually, without the support of a software package such as NVivo or Atlas.ti. Though we transcribed 51 videos, there was some similarity in the commentary across the dataset (i.e. wrestlers tended to use the same jokes, and certain attractions had a similar wrestling script across all performances). As a result, we felt the dataset was small and manageable enough to conduct rigorous manual coding using hard copies of the data and without spending the time, resources, and investment required to use a software package. In making this decision, we consulted the literature and ultimately used the recommendation by Basit (2003), who argued that deciding between manual and software coding should be “dependent on the size of the project, the funds and time available, and the inclination and expertise of the researcher,” (p. 143). We have updated the text beginning in line 355 to explain this reasoning, and to acknowledge the limitations of manual coding.

Quantitative results:
R3.8) Line 249: Check journal requirements, but it is not usual to start a sentence with a percentage written in numbers (eg ‘55%’ here). This will need checking throughout the manuscript.

We have converted numerical percentages to words in each instance where they occurred at the beginning of a sentence in the manuscript. Thank you for bringing this to our attention.

R3.9) Line 250: ‘gators’ rather than ‘alligators’ used here with no apparent reason for the difference. This also occurs elsewhere on the manuscript (eg line 267 ‘alligators’ but line 268 ‘gators’ – in the same sentence). Suggest using one term (alligators) consistently throughout the text.

We have changed all instances of ‘gator’ to ‘alligator’ and ‘gators’ to ‘alligators’ throughout the text, unless the term ‘gator’ was included in a direct quote or the name of a wrestling attraction. In line 232-233, we also made a note that the term ‘gator’ is a common colloquial term for alligators to avoid any confusion on the part of the reader.

R3.10) Line 292: preventing the animal from ‘slamming’ its mouth shut, or from closing it at all? Is the issue here the ‘slam’ (if not, this word seems inappropriate)?

Thank you for bringing this to our attention. Alligators tend to slam their mouths shut (versus closing their mouths gently) after having their jaws held open by wrestlers. We have changed the phrase “slamming its mouth shut” to “closing its mouth” in line 488 to avoid coming across as misleading.

Qualitative results:
R3.11) Line 308: the figure of 94% comes from anatomy and/or culture (ie as two different categories combined, such that each performance may only have one of the two but still be counted here) or where both anatomy and culture were mentioned in the same performance? Later (lines 347-348) 94% is cited just for anatomy and 41% for culture … confusing this a bit further. Clarity is needed.

We are very grateful for this suggestion, as originally, we calculated each sub-theme as a percentage of the entire data set (rather than a percentage within the broader themes they were coded under). This is likely what is causing the confusion, and we have made substantial edits to the section to adjust the percentages accordingly. The 94% figure indeed comes from Theme 2, Miscellaneous Education (which includes comments about anatomy and/or culture; as noted in the comment above, each performance may include one or both types of comments and still be included under Theme 2). However, the percentage of anatomy comments within Theme 2 should read as 100% (not 94%, as we originally wrote in the manuscript). Similarly, the percentage of culture comments should read as 44%, and not 41%. As such, we have updated the percentages in Table 2 (starting in line 382) and in the qualitative results section (beginning in line 507). We labeled all themes by number (i.e. in lines 517, 598, and 658) and noted which items were sub-themes (i.e. line 518, 601, 661) to help make the distinction more apparent for readers. Finally, to account for the fact that the percentages do not add up to 100%, we added a clarification in line 513: “Because wrestlers often addressed more than one theme and/or sub-theme in their commentary, the percentages throughout this section do not add up to 100%.”

R3.12) Line 342-343: Unclear sentence. Needs rewording.

We have edited the sentence, which now reads (beginning line 593): “Finally, none of the commentators noted whether their alligator wrestling attraction makes a monetary donation toward external conservation or protection initiatives, which may be another way to benefit ongoing conservation efforts.”

R3.13) Line 389: Safe to assume your international audience know what a ‘hickey’ is?

We have amended line 691 to clarify the definition of a hickey as a blemish caused by kissing or sucking on the skin. Thank you for pointing out that this definition is not universal!

Discussion:
R3.14) The numbered research questions referred to in the methods section are not revisited (at least not with reference to numbers) in the discussion. Conclusion is strong and relevant to the findings presented, but needs basing in the aim/research questions which should have been clearly identified at the outset.

We amended the text to remove the reference to numbered research questions in line 270, based on an earlier suggestion. As such, we feel that the conclusion more strongly addresses the overarching aim that is now presented at the outset of the manuscript.

Overall:
R3.15) The content is very interesting and the manuscript well written.

We are extremely grateful for this encouraging assessment, and we want to reiterate to the reviewer that their comments have been invaluable for helping us enhance the quality of our manuscript.

R3.16) A definition of ‘conspecific’ might be warranted, rather than assuming all readers will be familiar with this term that is used often in the paper. Lines 261-263 describe this without explaining at that point that conspecifics is the topic?

We have added a definition for conspecifics the first time it is used in the introduction (lines 110-111) to ensure the definition is clear throughout the manuscript.

R3.17) ‘Stunts’ and ‘tricks’ seem to be used interchangeably throughout the manuscript. Are they the same thing, or different? If the former, I suggest using just one term consistently throughout. If the later, then an explanation of the difference to avoid confusion would be beneficial.

The terms are meant to be used interchangeably, so we have removed all references to “tricks” and used stunts throughout the manuscript.

R3.18) Similarly, sometimes ‘alligator wrestling matches’ is used and sometimes more simply ‘alligator wrestling’. Are they the same thing, or different? If the former, use one term consistently throughout. If the later, then explain the difference to avoid possible confusion.

We agree that this gets slightly confusing, especially as we have used several terms interchangeably with matches, i.e. performances and shows. Per this suggestion, we have removed all references to alligator wrestling “matches” in the manuscript, using only the term “alligator wrestling” wherever possible. In several cases where we needed to refer to a venue or facility that offers alligator wrestling to the public, we have used the consistent term “alligator wrestling attraction” or simply “wrestling attraction” or “attraction” (for example in line 234). Similarly, in instances where we needed to refer to a specific alligator wrestling performance (versus discussing alligator wrestling as a broader activity), we have consistently used the term “alligator wrestling performance” or simply “performance” (for example in line 309). We chose the term performance because we do not want to mislead readers into thinking that alligator wrestling performances are “matches,” which could make it appear that the “competitors” have equal standing. In reality, the alligators can never “win” during an alligator wrestling performance, so we felt this term was the most accurate to use. Finally, in instances where we had to refer to the physical location where the alligator wrestling occurred, we used the term “performance arena” or simply “arena” (for example line 386). For clarity, we have also defined what we mean by performance arena in line 274-275 of the manuscript.

R3.19) The major issue to clarify (see above) is the research questions.

We appreciate your supportive and insightful suggestions. We have clarified the overarching aim of the manuscript and removed the reference to numbered research questions per the comments above, and we are very grateful to the reviewer for helping us add consistency and clarity to strengthen our arguments.
